# Comparison of Branched and Linear Perfluoropolyether Chains Functionalization on Hydrophobic, Morphological and Conductive Properties of Multi-Walled Carbon Nanotubes

**DOI:** 10.3390/nano8030176

**Published:** 2018-03-19

**Authors:** Maurizio Sansotera, Sadaf Talaeemashhadi, Cristian Gambarotti, Carlo Pirola, Mariangela Longhi, Marco A. Ortenzi, Walter Navarrini, Claudia L. Bianchi

**Affiliations:** 1 Dipartimento di Chimica, Materiali e Ingegneria Chimica, Politecnico di Milano, via Mancinelli 7, I-20131 Milano, Italy; sadaf.talaeemashhadi@chem.polimi.it (S.T.); cristian.gambarotti@polimi.it (C.G.); walter.navarrini@polimi.it (W.N.); 2 Consorzio Interuniversitario Nazionale per la Scienza e Tecnologia dei Materiali (UdR-PoliMi), via G. Giusti, 9, 50121 Firenze, Italy; 3 Dipartimento di Chimica, Università degli Studi di Milano, via Golgi 19, I-20133 Milano, Italy; carlo.pirola@unimi.it (C.P.); mariangela.longhi@unimi.it (M.L.); marco.ortenzi@unimi.it (M.A.O.); 4 Consorzio Interuniversitario Nazionale per la Scienza e Tecnologia dei Materiali (UdR-UniMi), via G. Giusti, 9, 50121 Firenze, Italy

**Keywords:** carbon nanotubes, functionalization, surface chemistry, superhydrophobicity

## Abstract

The functionalization of multi-walled carbon nanotubes (MW-CNTs) was obtained by generating reactive perfluoropolyether (PFPE) radicals that can covalently bond to MW-CNTs’ surface. Branched and linear PFPE peroxides with equivalent molecular weights of 1275 and 1200 amu, respectively, have been thermally decomposed for the production of PFPE radicals. The functionalization with PFPE chains has changed the wettability of MW-CNTs, which switched their behavior from hydrophilic to super-hydrophobic. The low surface energy properties of PFPEs have been transferred to MW-CNTs surface and branched units with trifluoromethyl groups, CF_3_, have conferred higher hydrophobicity than linear units. Porosimetry discriminated the effects of PFPE functionalization on meso-porosity and macro-porosity. It has been observed that reactive sites located in MW-CNTs mesopores have been intensively functionalized by branched PFPE peroxide due to its low average molecular weight. Conductivity measurements at different applied pressures have showed that the covalent linkage of PFPE chains, branched as well as linear, weakly modified the electrical conductivity of MW-CNTs. The decomposed portions of PFPE residues, the PFPE chains bonded on carbon nanotubes, and the PFPE fluids obtained by homo-coupling side-reactions were evaluated by mass balances. PFPE-modified MW-CNTs have been characterized by X-ray photoelectron spectroscopy (XPS), scanning electron microscopy (SEM), thermogravimetric analysis (TGA), static contact angle (SCA), surface area, and porosity measurements.

## 1. Introduction

Carbon nanotubes (CNTs) are chemically stable and mechanically resistant due to their rolled-up graphene planes with extensively delocalized π electron systems [1]. However, the lack of solubility in any solvent and the difficulty in manipulation have imposed great limitations to the application of CNTs [2,3,4,5,6,7,8,9]. In order to improve their ability to disperse and to facilitate their manipulation, many researchers have attempted the functionalization of CNT surface [10,11]. Several sidewall derivatization methods were developed by using fluorine [12], carbenes [13], azomethine ylides [14], and/or organic radicals [15]. Among these approaches, CNTs fluorination has been identified as one of the most effective chemical methods to modify and control CNTs physicochemical properties [12,16,17,18]. There are several methods to fluorinate CNTs including fluorinating agent decomposition [19,20], direct fluorination with elemental fluorine, either static or dynamic [17,21], plasma fluorination with fluorocarbons [22,23], and catalytic fluorination [24]. The introduction of fluorine, perfluoroalkyl, and other fluorine-containing moieties into organic compounds can deeply change the physical, chemical, and biological properties of the starting material [25,26]. Therefore, CNTs functionalization with fluorinated groups can be considered a suitable technique for transferring the unique features of fluorinated materials like low surface energy as well as high thermal and chemical stability to the carbon-based matrix [27,28,29,30]. Perfluoropolyethers (PFPEs) are liquid fluoropolymers embodying supplementary properties such as high gas permeability and high molecular mobility in addition to those of fluorinated materials [27]. PFPE-functionalization can be performed by employing several functional PFPE reagents, but the majority of them contains hydrogenated spacers lack the same thermal and chemical stability of fluoromaterials [28,31,32]. To this aim, PFPE peroxides are advantageous reagents since their thermolysis generates highly reactive perfluorinated radicals, which are able to bond directly with MW-CNTs sidewall without employing detrimental spacers [33]. Therefore, PFPE-functionalization with perfluorinated peroxides can achieve the production of stable superhydrophobic MW-CNTs, which, due to their extraordinary low moisture, find applications in several fields like water management, water-oil separation, self-cleaning, self-lubrication, water repellent surfaces, micro-reactors, and microfluidic systems [34,35,36,37,38,39].

In the present work, the sidewall functionalization of MW-CNTs with branched and linear PFPE moieties was studied by focusing on the effects ascribable to branching and linearity of PFPE residuals. The covalent linkage of PFPE chains on the MW-CNTs surface was obtained by thermal decomposition of different PFPE peroxides in the presence of MW-CNTs. It was, therefore, expected that the typical properties of fluorinated materials in particular the low surface energy could be transferred to the CNT surface [40]. The static and dynamic contact angle measurements with water were studied in order to characterize the super hydrophobic properties of the functionalized CNT samples. The conductive properties of MW-CNTs and their surface elemental compositions were studied by conductivity measurements and by X-ray photoelectron spectroscopy (XPS), respectively. Morphology changes and surface area variations on MW-CNTs were characterized by scanning electron microscopy (SEM), surface area, and porosity measurements. The thermal stability of the PFPE-functionalized MW-CNTs has been evaluated by thermal gravimetric analysis (TGA). The chemical functionalization with PFPE peroxides was also compared to the physical adsorption of inert PFPE fluids. A specimen of conductive MW-CNTs was also directly fluorinated with elemental fluorine in mild conditions. Its properties were analyzed in order to observe differences and analogies with MW-CNTs functionalized by PFPE chains.

## 2. Results

### 2.1. Functionalization Mechanism

**Figure 1:**
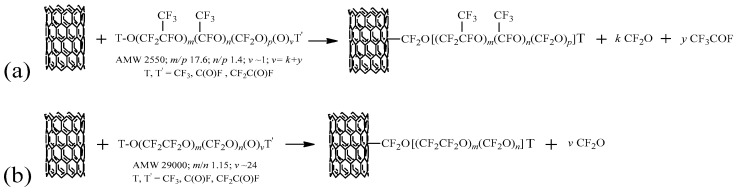
Functionalization reaction of MW-CNTs with branched (**a**) and linear (**b**) PFPE peroxides.

The thermal-induced homolytic cleavage of O–O bonds in PFPE peroxides synthesizes highly reactive radicals with PFPE structures, which can directly bond to carbonaceous structures without thermally and chemically detrimental spacers [27,41,42,43]. In general, the chemical treatments with PFPE peroxides lead to the formation of non-peroxidic PFPE layers covalently linked to the carbon-based surface (see Figure 1). The thermal decomposition of PFPE peroxide generates oxyradicals that produce carbon-centered PFPE radicals by β-scission reaction. The carbon-centered PFPE radicals participate to the functionalization mechanism in which the graphene layers of MW-CNTs act as radical scavengers performing the chemical linkage of PFPE chains to MW-CNTs sidewalls [34]. At this stage, sp^2^ carbon atoms change the hybridization to sp^3^ due to the formation of covalent bonds between PFPE radicals and cyclic moieties in graphene sidewalls of MW-CNTs. Carbonyl difluoride, COF_2_, and acetyl fluoride, CF_3_COF, are generated as side-products of the thermolysis of branched PFPE peroxide (see Figure 1a). As shown in Figure 1b, if the PFPE peroxide is linear, only COF_2_ can be produced. The homo-coupling of PFPE radicals competes with MW-CNTs functionalization by forming not-bonded PFPE fluids [27].

The sidewall functionalization of MW-CNTs was performed employing two different PFPE peroxides with similar equivalent molecular weights (EMWs) including a branched PFPE peroxide and a linear PFPE peroxide (see Figure 1). MW-CNTs were suspended in solutions of the corresponding PFPE peroxide dissolved in CF_3_OCFClCF_2_Cl and the solvent was then evaporated for depositing molecules of the peroxidic reagent on MW-CNTs surface. The peroxides were thermally decomposed and, therefore, reactive PFPE radicals were produced in the near proximity of MW-CNTs surface. Samples **I-BP50** and **II-LP50** were prepared by using branched and linear PFPE peroxides, respectively (see Table S1 in the Supplementary Materials).

The fluorination of MW-CNTs was performed in mild conditions in a below-atmospheric environment of F_2_ at temperatures ranging from 25 to 80 °C (sample **III-F**; Table S2 in the Supplementary Materials). The fluorination of MW-CNTs with F_2_ is usually associated with a transition from sp^2^ to sp^3^ in carbon hybridization by creating covalent C–F bonds [44]. Therefore, it was expected that fluorine atoms were covalently bonded to the carbon-based surface and to superficial zone of MW-CNTs. The bulk of the sample was preserved and only surface fluorinated sp^3^ carbon systems were generated.

**Figure 2:**
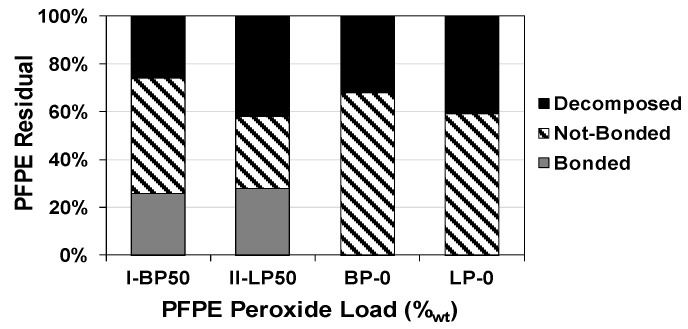
Bonded, non-bonded, and decomposed PFPE residuals after MW-CNTs functionalization compared to decomposition of pure PFPE peroxides.

The portions of linked, non-linked, and decomposed PFPEs after each functionalization were determined and were expressed as a percentage referred to the corresponding initial load of PFPE peroxide (see Figure 2 and Table S3 in the Supplementary Materials). The PFPEs chains bonded to MW-CNTs were deduced by a mass balance with the other PFPE fractions and resulted equally to approximately 26% in **I-BP50** and 28% in **II-LP50**. The portion of linked branched PFPE resulted in roughly similar components to that of linear PFPE because both PFPE peroxides, branched and linear, had comparable EMWs (1275 and 1200 g/eq, respectively).

Since the carbon-based structure of MW-CNTs is completely stable at the temperatures of the functionalization procedure, it was possible to evaluate the portions of peroxidic PFPE that decomposed to carbonyl difluoride, CF_2_O, and acetyl fluoride, CF_3_C(O)F, by weighting MW-CNTs after the thermal treatment. The N_2_-inerted reaction environment minimized the production of other by-products ascribable to the presence of oxygen or moisture. The decomposed portions measured during the functionalization of the branched sample, **I-BP50**, and linear sample, **II-LP50**, reached around 26% and 42%, respectively. In pure samples of PFPE peroxides, **BP-0** and **LP-0**, the thermolysis decomposed around 32% and 41% of the initial load, respectively. Therefore, the decomposed portions in **BP-0** and **I-BP50** were lower than those in **LP-0** and **II-LP50**. This difference can be attributed to the presence of a not-peroxidic PFPE content in the starting material of the branched peroxide.

The PFPE radicals that homo-coupled and unachieved the functionalization, formed inert PFPE fluids which were recovered at the end of the treatment and weighted for each sample by evaporating the washing solvents. Referring to the initial load of the corresponding PFPE peroxide, the PFPE fluids due to radical homo-coupling were 48% for **I-BP50** and 30% for **II-LP50**. The results indicated that the lower the peroxidic content in the polymeric reagent, the higher the relative amount of final PFPE fluids not linked to MW-CNTs sidewalls.

### 2.2. Effect on Wettability

PFPE-functionalization and fluorination modified the wettability of MW-CNTs from the initial hydrophilic surface properties to the exceeding super-hydrophobicity threshold. SCA measurements on pellets of pure MW-CNTs revealed that the water droplets were adsorbed in a few second (2–4 s) by this carbonaceous matrix because of the porous structure of MW-CNTs bundles. Conversely, the SCA super-hydrophobicity threshold (150°) was exceeded on both branched and linear PFPE-functionalized samples and also on the fluorinated specimen. Moreover, measurements of contact angle hysteresis revealed values below 10° on all these samples (see Table 1). PFPE peroxides can cover the rough surface of MW-CNTs with highly hydrophobic fluorinated chains [41]. Branched PFPE chains of the functionalized surface have larger encumbrance due to trifluoromethyl groups, CF_3_, than linear PFPE moieties. Therefore, it was expected that the branched units can generate a higher hydrophobicity than linear units for the same reason that the presence of more electronegative fluorine atoms induces a more marked wettability lowering. The comparison between **I-BP50** and **II-LP50** confirmed this trend since SCA values of 174° and 159° were measured, respectively. The fluorination with elemental fluorine on sample **III-F** chemically modified the composition of MW-CNTs and a layer of carbon fluorides was generated on the carbon-based surface [45]. Therefore, the synthesis of fluorinated groups on the MW-CNTs surface conferred highly hydrophobic properties to sample **III-F** and a SCA value around 172° was recorded, which exceeded the super-hydrophobic threshold (see Table 1).

XPS data showed that the fluorine content on MW-CNTs surface appeared after PFPE-functionalization and fluorination (see Table 1). XPS analysis showed that in samples **I-BP50** and **II-LP50**, fluorine contents were 9.2 and 4.2 at %, respectively. The higher fluorine content due to functionalization with branched PFPEs can justify the more marked super-hydrophobicity observed on **I-BP50**. It is worth noting that, considering that branched and linear PFPE peroxides had similar EMWs, the branched reagent also achieved a higher functionalization degree. The branched PFPE peroxide, which is characterized by a low AMW of 2250 amu, functionalized the carbon-based surface and diffused toward the reactive sites into the inner pores of MW-CNTs aggregates. The macromolecular length of linear PFPE peroxide, related to AMW around 29,000 amu, probably hindered its internal diffusion. Due to the high mobility and reactivity (even in mild conditions) of elemental fluorine, the highest fluorine content was measured on sample **III-F** (14.2 at %). The oxygen content on the surface of pure MW-CNTs (1.3 at %) revealed the presence of several oxygenated functions such as alcohols, carbonyls, and carboxyls, which are generally observed. After PFPE-functionalization as well as after fluorination a general increase in oxygen content on MW-CNTs surface was measured. The oxygen contents in samples **I-BP50** and **II-LP50** were 2.1 and 2.4 at %, respectively, and were equated stoichiometrically, which is based on the fluorine content due to the linkage of the PFPE chains (see Table 1). On sample **III-F**, an oxygen increase was observed and it can be ascribed to the presence of oxygen traces or moisture in the reaction environment during the fluorination reaction. The absence of XPS signals due to chlorine atoms in samples **I-BP50** and **II-LP50** evidenced the complete removal of the solvent CF_3_OCFClCF_2_Cl by vacuum treatments at high temperature during the functionalization treatment. Thus, the fluorine content was due to PFPE-functionalization on MW-CNTs surface and cannot be attributed to adsorbed solvent.

### 2.3. Effect on Morphology

BET analyses revealed that the morphology of MW-CNTs (389 m^2^/g) was separately influenced by functionalization with branched PFPE peroxide or with linear PFPE peroxide as well as by fluorination with elemental fluorine (see Table 1). In sample **I-BP50**, PFPE-functionalization with branched chains generated a decrease in the surface area to 231 m^2^/g. In sample **II-LP50**, the covalent linkage of linear PFPE chains changed remarkably the hydrophobic properties of MW-CNTs, but the values of the surface area remained high around 308 m^2^/g. Direct fluorination with F_2_ in mild conditions (sample **III-F**) also decreased MW-CNTs surface area to 277 m^2^/g. These data indicate that PFPE-functionalization with branched chains influenced the morphology of native MW-CNTs, which aggregate more than the treatments with linear PFPE peroxide or with F_2_.

The physical adsorption of PFPE fluids onto the MW-CNTs also generated a variation in the surface properties (see Preparation of Comparative Examples in the Supplementary Materials). After the physisorption of branched and linear PFPE fluids, fluorine contents of 3.8 and 4.7 at % were observed, respectively, and the surface area decreased to 243 and 229 m^2^/g, respectively (see Tables S4 and S5). Contact angle values of 148° and 170° were measured, respectively, revealing that the typical hydrophobic properties of the fluorinated chains were conferred to the MW-CNTs surface (see Table S5). However, washings with fluorinated solvent increased the surface area and removed the hydrophobic properties because no chemical linkage of the fluorinated chains occurred in PFPE physisorption (see Table S5). In fact, after washings, no fluorine content on the MW-CNTs surface was detected by XPS analysis as demonstrated by the removal of physisorbed PFPE fluids (see Table S4). Conversely, surface properties like surface composition, surface area, and moisture of samples **I-BP50**, **II-LP50** were permanently stable even after they were washed continuously for 24 h with pure fluorinated solvent by using a Soxhlet extractor (Tables S4 and S5).

**Figure 3:**
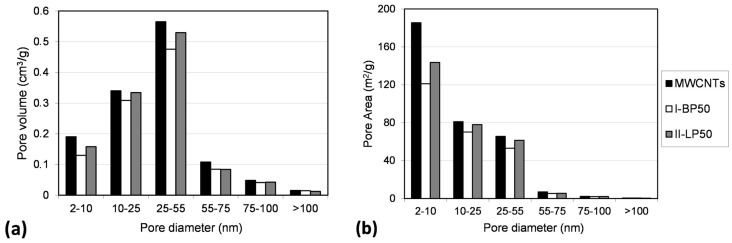
Pore volumes (**a**) and pore areas (**b**) of samples before (MW-CNTs) and after PFPE-functionalization with branched (**I-BP50**) and linear (**II-LP50**) peroxides.

The characterization of pore volume, pore area, and pore size distribution provided quantitative and detailed information about the pore structure of MW-CNTs samples (see Figure 3a,b). It is worth noticing that Barrett-Joyner-Halenda (BJH) method, which is based on the macroscopic Kelvin equation, cannot provide reliable information about the microporous structure. Therefore, only mesoporous and macroporous structures were considered [46]. As shown in Figure 3, volumes and areas of the mesoporous structure in the ranges between 2 to 10 nm, 10 to 25 nm, and 25 to 55 nm decreased significantly after PFPE-functionalization. In particular, volumes and areas of mesopores in these ranges decreased more in sample **I-BP50** than in sample **II-LP50**. This effect can be attributed to the AMW of branched PFPE peroxide, which is significantly lower than that of linear PFPE peroxide. Therefore, due to the relatively short length of its macromolecules, the branched reagent diffused towards the reactive sites located in the internal pores of MW-CNTs aggregates. In the region of the macroporous structure, which include the ranges between 55 to 75 nm, 75 to 100 nm, and more than 100 nm, pore volumes and pore areas slightly decreased in both samples **I-BP50** and **II-LP50** without evident differences between branched and linear PFPE-functionalization.

The porosity data were normalized by the surface area and the pore distribution was calculated for each sample (see Figure S1). These data revealed that the decrease in the pore structure due to PFPE-functionalization mostly influenced the mesoporosity in the range between 2 to 10 nm. In fact, both branched and linear PFPE peroxides have EMW that fits with PFPE chains, which can enter into MW-CNTs mesopores with this size. The other part of the pore distribution was mainly preserved after PFPE-functionalization, which suggests that PFPE-grafting on MWCNTs surface was almost homogeneous.

**Figure 4:**
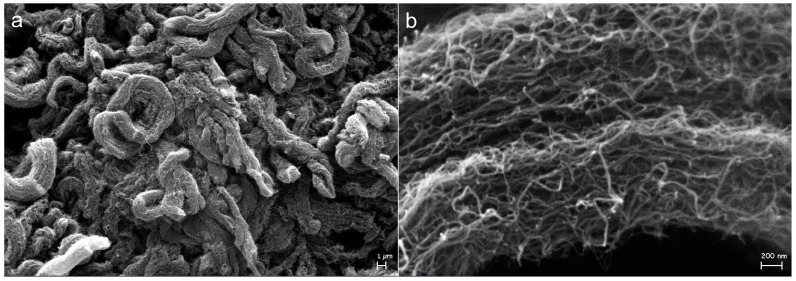
SEM micrographs of native MW-CNTs: 2.5 (**a**) and 50 kx (**b**).

**Figure 5:**
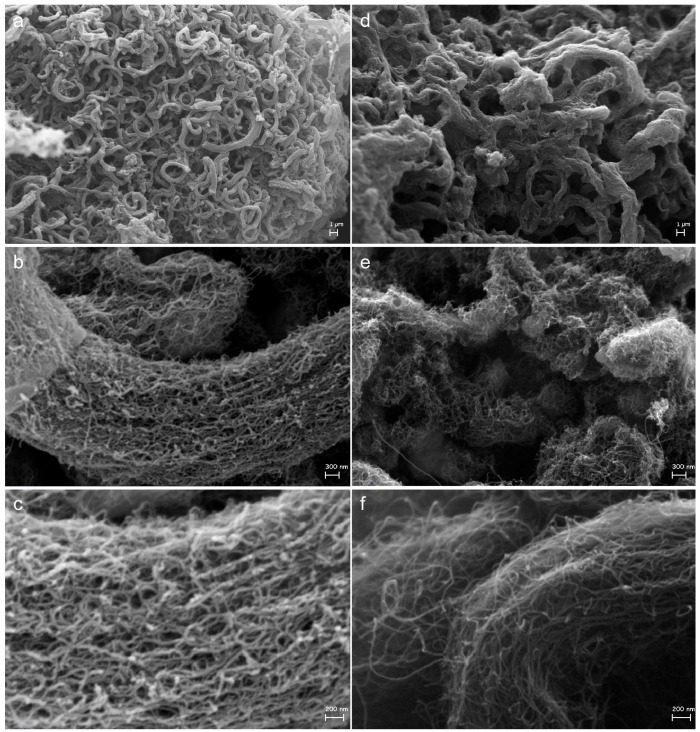
SEM micrographs of MW-CNTs after functionalization with branched (**I-BP50**) and linear (**II-LP50**) PFPE peroxides: 2.5 (**a**,**d**), 50 (**b**,**e**) and 100 kx (**c**,**f**).

MW-CNTs naturally align into “ropes” held together by π-stacking forces (see Figure 4a). The high magnification images of native MW-CNTs showed the aggregation of MW-CNTs in disordered bundles and the remaining interstitial spaces (see Figure 4b). The typical rope-like alignment of MW-CNTs was preserved also on sample **I-BP50**, in which the layer of branched PFPE covered almost homogeneously MW-CNTs bundles (see Figure 5a) forming only few PFPE aggregates (Figure 5b,c). The polyperoxidic structure of PFPE peroxides allowed the linkage in a row of several connected PFPE chains, which gradually grew the entire PFPEs layers on MW-CNTs surface [47]. Similarly, after treatment with linear PFPE peroxide (see Figure 5d–f) as well as with elemental fluorine (Figure S2 in the Supplementary Materials), the bundled aggregation was overall maintained and avoided MW-CNTs disaggregation.

### 2.4. Effect on Conductive Properties

**Figure 6:**
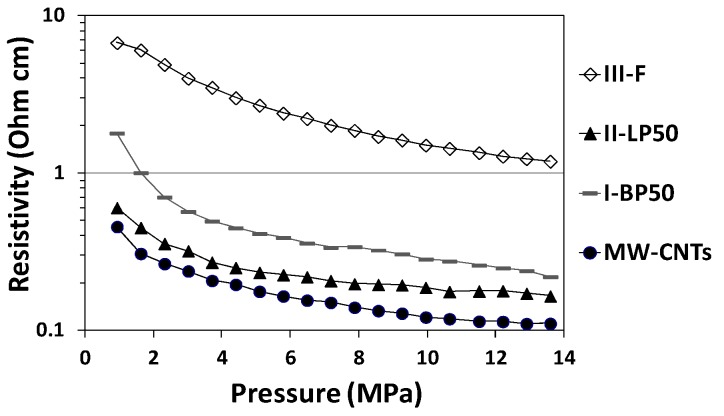
Resistivity (in logarithmic scale) of conductive MW-CNTs at different pressures before (MW-CNTs) and after PFPE-functionalization with branched (**I-BP50**) and linear (**II-LP50**) peroxides and after fluorination with F_2_ (**III-F**).

MW-CNTs resistivity was evaluated in function of the applied pressure for characterizing the electrical properties before and after PFPE-functionalization as well as after fluorination with F_2_ (see Figure 6 and, for detailed data, Table S6). The compacting pressure due to the loadings caused resistivity changes ascribable to deformation of the carbon-based porous structure. Additionally, compression increases local contact forces between MW-CNTs for a better contact, which leads to a decrease in the contact resistance between crossing nanotubes [48,49]. For native MW-CNTs (see Figure 6), the electrical resistivity varied in the range 0.1–0.5 Ω∙cm. After PFPE-functionalization with branched and linear peroxides, the resistivity values slightly increased in the range of conductive materials (see Figure 6). The ohmic increment was due to the typical non-conductive properties of PFPEs chains endowing MW-CNTs. The electrical resistivity of sample **I-BP50** was higher when compared to that of sample **II-LP50** because of the higher functionalization degree obtained by the branched reagent. As expected, the highest values of electrical resistivity were measured on F_2_-fluorinated MW-CNTs (**III-F**): 6.7 Ω∙cm at 0.9 MPa and 1.1 Ω∙cm at 13.6 MPa. In addition, F_2_ fluorination caused the covalent linkage of fluorine atoms to carbon atoms directly on MW-CNTs surface and into the sub-superficial zone of the particles, which changed the sp^2^ state of the carbon-based layers of the conductive π-electron system in sp^3^ hybridization [50]. However, the resistivity values at high applied pressures may suggest that the conductive properties in the bulk of F_2_-fluorinated MW-CNTs (**III-F**) were partially preserved.

### 2.5. Effect on Thermal Stability

**Figure 7:**
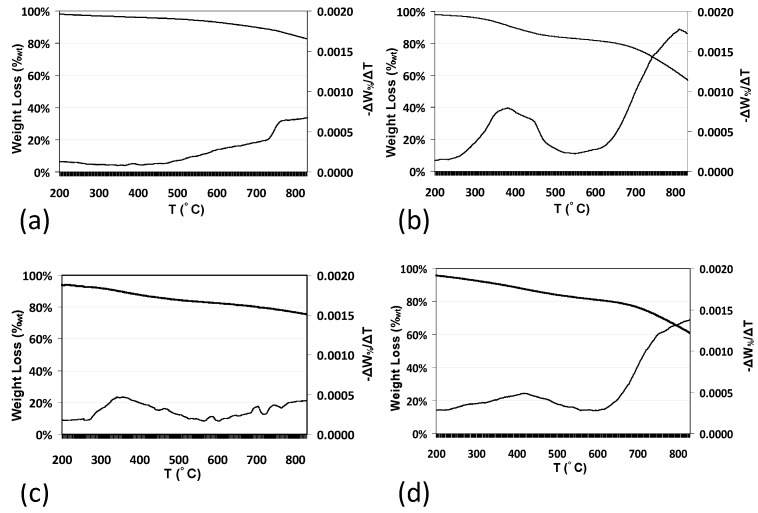
TGA thermograms of MW-CNTs under N_2_ before (**a**) and after PFPE-functionalization with branched (**b**) and linear (**c**) peroxides and after fluorination with F_2_ (**d**).

The thermal stability of PFPE-functionalized and fluorinated MW-CNTs was evaluated by TGA analysis (see Figure 7). TGA curve of native MW-CNTs revealed that a weak degradation began at around 500 °C and almost 80 wt % of the MW-CNTs mass remained stable up to 800 °C (see Figure 7a). MW-CNTs functionalized with branched (**I-BP50**) and linear (**II-LP50**) PFPE peroxides (see Figure 7b,c) showed weight losses in two separated steps including the first weight loss ranging from approximately 240–490 °C and it was due to the degradation of PFPE chains grafted on MWCNTs sidewall. A second weight loss started after 500 °C and it can be ascribed to the degradation of modified parts of carbon nanotubes. For the fluorinated sample **III-F**, a weight loss of around 12 wt % occurred between 235 and 500 °C due to defluorination of MWCNTs (see Figure 7d).

## 3. Materials and Methods

### 3.1. Materials

MW-CNTs (Nanocyl 7000, Sambreville, Belgium) prepared by the supplier through CVD process and were characterized by an average diameter of 9.5 nm and average length of 1.5 μm. Branched and linear PFPE peroxides are non-commercial Fomblin^®^ peroxides (Solvay Specialty Polymers, Bollate, Italy). They were synthesized by photooxidative polymerization of tetrafluoroethylene (TFE) and hexafluoropropene (HFP) [45,51] and were kindly provided for this research. Two comparative samples were also prepared by using Fomblin^®^ YHVAC 18/8 and Fomblin^®^ M03, which both do not contain peroxidic moieties. The chemical characteristics of the PFPE compounds are reported below.

- branched PFPE peroxide with general formula TO[CF_2_CF(CF_3_)O]_m_[CF(CF_3_)O]_n_(CF_2_O)_p_(O)_v_T: average molecular weight (AMW) around 2550 amu, equivalent molecular weight (EMW) around 1275 g/eq, ratio between perfluoro-*iso*-propylene oxide (C_3_, i.e., (CF_3_)CFCF_2_O and CF_2_CF(CF_3_)O randomly distributed), perfluoro(methyl)methylene oxide (C_2_, i.e., CF(CF_3_)O) and perfluoromethylene oxide (C_1_, i.e., CF_2_O) units 17.6:1.4:1, peroxidic content 0.286 wt % determined by iodometric titration [52];
- branched PFPE fluid: Fomblin^®^ YHVAC 18/8 by Solvay Specialty Polymers Inc., MWA around 2800 amu, ratio between perfluoro-*iso*-propylene oxide, (C_3_, i.e., CF_2_CF(CF_3_)O and CF_2_CF(CF_3_)O randomly distributed), and perfluoromethylene oxide, (C_1_, i.e., CF_2_O), units around 15, no peroxidic moieties along the polymer chain;
- linear PFPE peroxide with general formula TO(CF_2_CF_2_O)_m_(CF_2_O)_n_(O)_v_TO: AMW around 29,000 amu, EMW around 1200 g/eq, ratio between perfluoroethylene oxide, (C_2_, i.e., CF_2_CF_2_O), and perfluoromethylene oxide, (C_1_, i.e., CF_2_O), units (*m/n*) around 1.15, peroxidic content 1.3 wt %;
- linear PFPE fluid: Fomblin^®^ M03 by Solvay Specialty Polymers Inc. (Woodburn, OR, USA), AMW around 4000 amu, ratio between perfluoroethylene oxide, (C_2_, i.e., CF_2_CF_2_O) and perfluoromethylene oxide, (C_1_, i.e., CF_2_O), units (*m/n*) around 1, no peroxidic moieties along the polymer chain (*v* = 0).

CF_3_OCFClCF_2_Cl (b.p. 40–41 °C) was employed as a solvent because of its absence of reactivity in the presence of PFPE peroxides as well as of PFPE radicals.

Pure elemental fluorine, F_2_, stored in an appropriate cylinder (8 bar, Solvay Fluor) was employed for direct fluorination of MW-CNTs.

### 3.2. PFPE-Functionalization of MW-CNTs

MW-CNTs (6 g) were suspended in a solution of PFPE peroxide (3 g) dissolved in CF_3_OCFClCF_2_Cl (150 mL) as fluorinated solvent. The PFPE peroxidic MW-CNTs suspension was homogenized by intense magnetic stirring and sonication (5 min). Thereafter, the solvent was completely evaporated at 40 °C. PFPE peroxides were thermally decomposed by increasing (5 °C/h) stepwise the temperature from 150 up to 200 °C, which later remained at 200 °C for 2 h. At the end of the thermal treatment, the residue of functionalized MW-CNTs was recovered, filtered on a PTFE-membrane with pore size of 0.45 μm (Sartorius Stedim Biotech, Göttingen, Germany) and washed three times with CF_3_OCFClCF_2_Cl (150 mL) and three times with deionized water (150 mL). PFPE-functionalized MW-CNTs were finally dried under vacuum (0.01 mmHg) at 200 °C for 24 h. Samples **I-BP50** and **II-LP50** were prepared by using branched and linear PFPE peroxide, respectively (see Table S1 in the Supplementary Materials).

PFPE-functionalization was compared to direct fluorination of MW-CNTs with F_2_. A fluorine pressure of around 100 mbar was introduced in the reactor for 15 min and, thereafter, the reactor was evacuated forcing all the gases to a soda lime trap. This procedure was repeated eight times at room temperature and two times at 80 °C. The conditions of the fluorination reaction (sample **III-F**) were reported in Table S2 in the Supplementary Materials.

Each sample of MW-CNTs was weighted before and after the thermal treatment in order to determine the portion of peroxidic PFPE that decomposed to carbonyl difluoride, CF_2_O, and acetyl fluoride, CF_3_C(O)F, during the thermolysis, which assumed a complete stability of CNTs matrix until 200 °C. The portion of PFPE that homo-coupled, shirking MW-CNTs functionalization, was evaluated by weighting the PFPE residue, which was obtained by evaporating the washing solvents. The portion of linked PFPE was obtained as the difference. The portions of linked, non-linked, and decomposed PFPEs were expressed as percentage referred to the corresponding initial load of PFPE peroxide.

### 3.3. Characterizations

DSA100 Series Instrument (Kruss, Hamnburg, Germany) equipped with DSA1 software (version 1.29.1.1, Kruss, Hamnburg, Germany) was employed for contact angle measurements. Pellets of MW-CNTs samples (5–20 mg) were prepared by press molding at room temperature under a load of 7000 kg/cm^2^ for a few minutes (3–5 min). Water droplets were deposited on MW-CNTs surface for SCA measurements. Drop volumes for advancing and receding contact angles were in the range from 8 to 20 μL. The hysteresis was calculated by subtracting the measured advancing contact angles with the measured receding contact angles.

An M-probe spectrometer (Surface Science Instrument, SSI, Mountain View, CA, USA) was used for X-ray photoelectron spectroscopy. The spectra were obtained by a monochromatic X-ray emission of A1 Kα radiation (1486.6 eV). A spot size of 200 × 750 µm and pass energy of 29 eV were used. 1 s level hydrocarbon-contaminant carbon was taken as the internal reference at 284.6 eV.

The specific surface areas were measured by N_2_ adsorption and calculated on the basis of Brunauer-Emmett-Teller (BET) theory. The N_2_ adsorption allowed for the determination of the total microporous surface area by means of the *t*-plot method. Pore volumes and pore areas were determined as a function of the pore diameter in the mesoporous and macroporous ranges by using the BJH method. Before analysis, sample were pre-treated under vacuum (0.1 mmHg) at 130 °C for 2.5 h. A Micromeritics TriStar II 3020, managed by the software TriStar II 3020 V1.03 (Norcross, GA, USA), was used for BET analysis and porosimetry.

Scanning electron microscope (Zeiss EVO–50, Zeiss, Thornwood, NY, USA), working distance 8.0 mm, beam current 100 pA, acceleration voltage 20.00 kV) was employed to analyze the morphology of MW-CNTs samples. Microscopy was performed on bare samples without deposition of a conductive layer.

The electrical resistivity measurements at different applied pressures (0.9–13.6 MPa) were performed by using a tailor-made apparatus assembled according to literature description [53]. The resistance (*R*) values were measured by using an AOIP OM21 Micro-ohmmeter (AOIP, Paris, France) and were converted in resistivity (*ρ*) by applying Equation (1).

(1) $$\rho =\frac{R\cdot \pi \cdot d^{2}}{4l}=\frac{1}{\kappa }$$

in which *d* is the diameter of apparatus tube, *l* is the distance between the two pistons of the apparatus, and *k* is the specific conductivity. Measurements were repeated three times on each MW-CNTs sample at the same pressures in order to decrease the experimental error of the test.

The thermogravimetric analyses were performed with a THASS TGA XP-10 (Thass, Friedberg, Germany)) analyzer. The samples (approximately 10 mg) were heated from 200 to 830 °C at a rate of 10°/min under N_2_ flow.

## 4. Conclusions

PFPE-functionalization of MW-CNTs was obtained by thermal decomposition of branched and linear PFPE peroxides. The linkage of PFPEs chains conferred super-hydrophobic properties to MW-CNTs surface, which was proven by contact angle measurements. The comparison between branched and linear PFPE-functionalization revealed that the branched reagent achieved a higher functionalization degree than the linear reagent. Porosimetry showed that branched PFPE peroxide reached the reactive sites located in the internal MW-CNTs mesopores due to the relatively short length of its molecules while linear PFPE-functionalization mostly preserved MW-CNTs mesomorphology. Macropores were partially influenced by PFPE-functionalization without evident differences related to the use of branched or linear reagents. Moreover, the marked moisture decreasing in branched PFPE-functionalized MW-CNTs is also due to the presence of more electronegative fluorine atoms due to trifluoromethyl groups, CF_3_. The SEM images indicated that MW-CNTs maintained rope-like structures after functionalization and disaggregation was not observed. PFPE-functionalized MW-CNTs remained electrically conductive even if their moisture varied significantly. In summary, PFPE-functionalization of MW-CNTs can be employed for a controlled modification of surface and morphological properties of carbon nanotubes without detriment to their conductivity.

## Figures and Tables

**Table 1:** Static and hysteresis contact angle measurements with water, surface composition (at %), specific surface area, and micropore area of MW-CNTs before and after PFPE-functionalization (**I-BP50**, **II-LP50**) and fluorination (**III-F**).

Specimen	Contact Angle		Surface Composition ^1^ (at %)			Specific Surface Area ^2^ (m^2^/g)	Micropore Area ^3^ (m^2^/g)
	Static	Hysteresis	F	O	C		
MW-CNTs	n.s. ^4^	-	-	1.3	98.7	389	31
**I-BP50**	174°	4.0°	9.2	2.1	88.7	231	0
**II-LP50**	159°	5.3°	4.2	2.4	93.4	308	0
**III-F**	172°	4.2°	14.2	2.0	83.8	277	26

^1^ Determined by XPS. ^2^ Determined through BET theory. ^3^ Determined by *t*-plot method. ^4^ not stable. Water droplets are quickly adsorbed (2–4 s) into MW-CNTs pellets.

## Supplementary Materials

The following are available online at http://www.mdpi.com/2079-4991/8/3/176/s1, Table S1: Experimental conditions of MW-CNTs functionalization with branched and linear PFPE peroxides, Table S2: Experimental conditions of MW-CNTs fluorination with F_2_, Table S3: Weight evaluations for determination of linked, non-linked and decomposed portions of PFPEs in MW-CNTs functionalization with PFPE peroxides, Preparation of Comparative Examples—Physisorption of linear and branched PFPE Fluids on MW-CNTs, Table S4: Surface composition (at %) by XPS analysis referred to the comparative example, Table S5: Contact angle with water and BET surface area measurements referred to the comparative example, Table S6: Electrical resistivity at different pressures of MW-CNTs before and after PFPE-functionalization with branched and linear PFPE peroxides and after fluorination with F_2_, Figure S1: Normalized pore volumes (a) and pore areas (b) of the samples before (MW-CNTs) and after PFPE-functionalization with branched and linear peroxides (**I-BP50** and **II-LP50**), Figure S2: SEM micrographs of fluorinated MW-CNTs (**III-F**): 2.5 (a) and 100 kx (b).
