# Supplementary material for: Comparison of Branched and Linear Perfluoropolyether Chains Functionalization on Hydrophobic, Morphological and Conductive Properties of Multi-Walled Carbon Nanotubes
Source: Nanomaterials (Basel). 2018 Mar 19;8(3):176. doi: 10.3390/nano8030176 (PMC5869667; doi:10.3390/nano8030176)
Supplement: Supplementary file 1 [file nanomaterials-08-00176-s001.pdf]

nanomaterials

MDPI

Supplementary Materials

Comparison of Branched and Linear Perfluoropolyether Chains Functionalization on Hydrophobic, Morphological and Conductive Properties of Multi-walled Carbon Nanotubes

Table S1. Experimental conditions of MW-CNTs functionalization with branched and linear PFPE peroxides.

Specimen	m_c[a] (g)	m_p[b] (g)	V[c] (ml)	Thermal Decomposition	
				T (°C)	t (h)
**I-BP50**	6.1850	3.1445	150	150-200	6
**II-LP50**	6.1278	3.1177	150	150-200	6
**BP-0**	-	0.4635	150	150-200	6
**LP-0**	-	0.4544	150	150-200	6

[a] MW-CNTs weight. [b] weight of PFPE peroxide. [c] volume of solvent.

Table S2. Experimental conditions of MW-CNTs fluorination with F_2.

Specimen	m_c[a] (g)	M_F[b] (mmol)	Steps[c]	P[d] (mbar)	T[e] (°C)	t[f] (h)
**III-F**	0.46	17.70	10	100	25-80	2.5

[a] MW-CNTs weight. [b] elemental fluorine millimoles. [c] reaction steps. [d] reaction pressure. [e] temperature range.[f] reaction time.

Table S3. Weight evaluations for determination of linked, non-linked and decomposed portions of PFPEs in MW-CNTs functionalization with PFPE peroxides.

Specimen	m_c[a] (g)	m_p[b] (g)	PFPE					
			Bonded		Non-Bonded		Decomposed	
			(g)	(%)	(g)	(%)	(g)	(%)
**I-BP50**	6.1850	3.1445	0.8168	26	1.4992	48	0.8285	26
**II-LP50**	6.1278	3.1177	0.8772	28	0.9288	30	1.3117	42
**BP-0**	-	0.4635	-	-	0.3145	68	0.1490	32
**LP-0**	-	0.4549	-	-	0.2693	59	0.1856	41

[a] MW-CNTs weight. [b] weight of PFPE peroxide.

Preparation of Comparative Examples

Physisorption of linear and branched PFPE Fluids on MW-CNTs

The MW-CNTs (6 g) and the non peroxidic solution (150 mL) were introduced into a glass reactor. Two samples were prepared, where the amount of branched and linear PFPE fluids were 0.63 g and 0.65 g, respectively. The MW-CNTs suspensions were heated at 40°C until the complete evaporation of the solvent (*i.e.* $CF_3OCFClCF_2Cl$). The solid residues were finally dried under vacuum (0.01 mmHg) at 40°C for 24 hours. The effects of the physisorpion of the not peroxidic branched and linear PFPE fluids were evaluated by BET analysis and contact angle measurement. After these characterizations, the samples were washed three times with 150 ml of pure fluorinated solvent ($CF_3OCFClCF_2Cl$) and then three times with 150 ml of deionized water. Finally, they were washed

continuously for 24 h with pure fluorinated solvent ($CF_3OCFClCF_2Cl$) by means of a Soxhlet extractor. Thus the BET and contact angle characterizations were repeated and furthermore the elemental composition was determined by XPS analysis. For additional comparison, a portion of the samples **I-BP50** and **II-LP50** were washed continuously for 24 h with pure fluorinated solvent ($CF_3OCFClCF_2Cl$) with Soxhlet extractor and then characterized as described before.

Table S4. Surface composition (at%) by XPS analysis referred to the comparative example.

Samples	Amount (at%)		
	F	O	C
MW-CNTs	-	1.3	98.7
MW-CNTs with physisorbed branched PFPE fluid (before washings)	3.8	1.1	95.1
MW-CNTs with physisorbed branched PFPE fluid (after washings)	0.7	0.8	98.5
MW-CNTs with physisorbed branched PFPE fluid (after Soxhlet washings)	0.6	0.7	98.7
MW-CNTs with physisorbed linear PFPE fluid (before washings)	4.7	2.2	93.0
MW-CNTs with physisorbed linear PFPE fluid (after washings)	0.6	1.5	97.9
MW-CNTs with physisorbed linear PFPE fluid (after Soxhlet washings)	-	1.2	98.8
**I-BP50**	9.2	2.1	88.7
**I-BP50** (after Soxhlet washings)	8.8	2.9	88.3
**II-LP50**	4.2	2.4	93.4
**II-LP50** (after Soxhlet washings)	3.5	2.6	93.9

Table S5. Contact angle with water and BET surface area measurements referred to the comparative example.

Samples	Contact Angle	Surface Area (m^2/g)
MW-CNTs	n.s.[a]	389
MW-CNTs with physisorbed PFPE branched fluid (before washings)	148°	243
MW-CNTs with physisorbed branched PFPE fluid (after washings)	n.s.[a]	299
MW-CNTs with physisorbed branched PFPE fluid (after Soxhlet washings)	n.s.[a]	329
MW-CNTs with physisorbed linear PFPE fluid (before washings)	170°	229
MW-CNTs with physisorbed linear PFPE fluid (after washings)	n.s.[a]	283
MW-CNTs with physisorbed linear PFPE fluid (after Soxhlet washings)	n.s.[a]	292
**I-BP50**	174°	231
**I-BP50** (after Soxhlet washings)	169°	258
**II-LP50**	159°	308
**II-LP50** (after Soxhlet washings)	156°	323

[a] the droplet is not stable and is adsorbed in few seconds into the pellet.

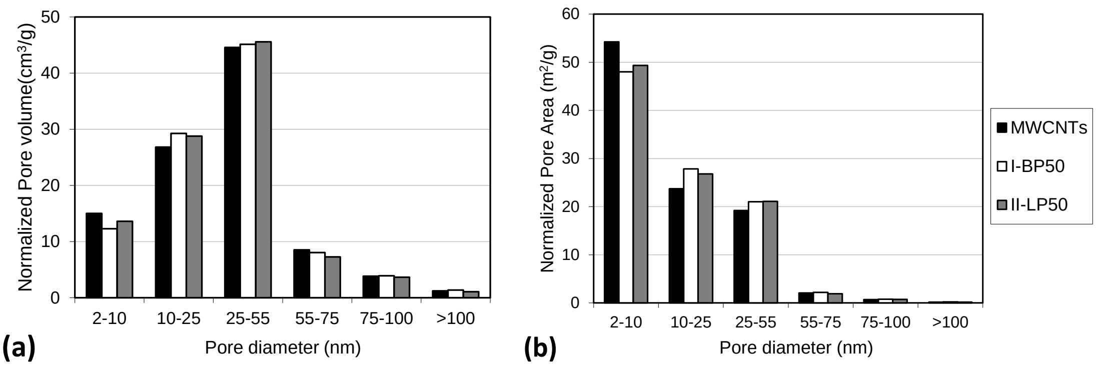

Figure S1. Normalized pore volumes (a) and pore areas (b) of the samples before (MW-CNTs) and after PFPE-functionalization with branched and linear peroxides (**I-BP50** and **II-LP50**).

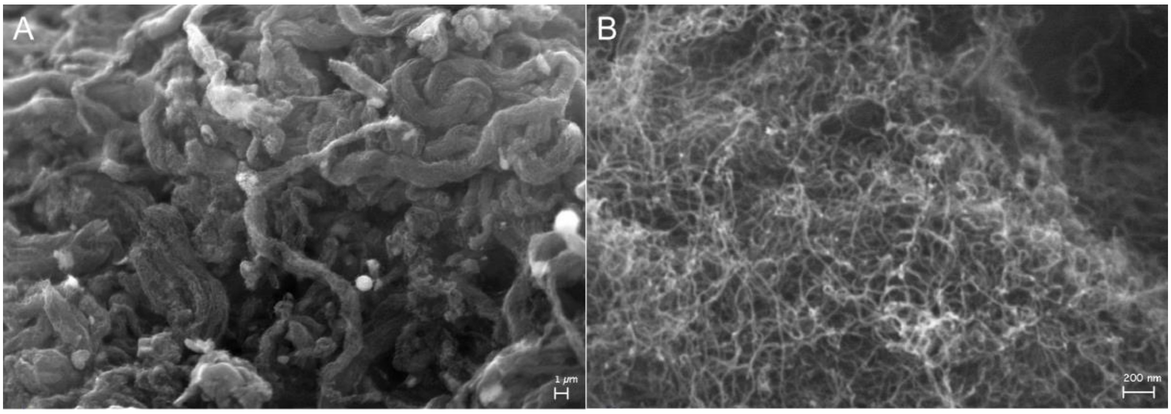

Figure S2. SEM micrographs of fluorinated MW-CNTs (**III-F**): 2.5 (a) and 100 kx (b).

Table S6. Electrical resistivity at different pressures of MW-CNTs before and after PFPE-functionalization with branched and linear PFPE peroxides and after fluorination with F_2.

**Pressure (MPa)**	**Resistivity (Ω · cm)**			
	**MW-CNTs**	**I-BP50**	**II-LP50**	**III-F**
**0.9**	0.46	1.800	0.6028	6.75
**1.6**	0.31	1.007	0.4507	6.06
**2.3**	0.27	0.704	0.3572	4.91
**3.0**	0.24	0.570	0.3209	4.01
**3.7**	0.21	0.496	0.2715	3.51
**4.4**	0.20	0.449	0.2507	3.01
**5.1**	0.18	0.413	0.2338	2.70
**5.8**	0.17	0.391	0.2260	2.41
**6.5**	0.16	0.359	0.2194	2.22
**7.2**	0.15	0.338	0.2075	2.01
**7.9**	0.14	0.341	0.1982	1.87
**8.6**	0.13	0.324	0.1960	1.71
**9.3**	0.13	0.306	0.1947	1.63
**9.9**	0.12	0.285	0.1880	1.51
**10.6**	0.12	0.276	0.1767	1.44
**11.5**	0.11	0.260	0.1780	1.35
**12.2**	0.11	0.253	0.1788	1.28
**12.9**	0.11	0.248	0.1722	1.24
**13.6**	0.11	0.221	0.1658	1.19
